# Can combining existing behavioral tools improve identification of infants at elevated likelihood of autism in the first year of life?

**DOI:** 10.1177/13623613241275455

**Published:** 2024-09-12

**Authors:** Meagan R Talbott, Gregory S Young, Sally Ozonoff

**Affiliations:** 1 Department of Psychiatry and Behavioral Sciences, MIND Institute, UC Davis Health, Sacramento, CA USA

**Keywords:** Autism spectrum disorders, early detection, infant sibling, screening

## Abstract

Identifying infants at elevated likelihood for autism and other developmental differences in the first year of life remains a significant challenge. This study explored associations between behavioral differences in infancy and developmental outcomes in toddlerhood. We conducted a secondary data analysis of 256 infants with an older autistic sibling followed from 6 to 36 months of age. We defined conceptually driven cutoffs for three behavioral measures collected at 6, 9, and 12 months and examined the utility of these measures, individually and in combination, in predicting 36-month outcome classification (typical development or developmental concerns, including autism). Overall, relatively few infants met multiple measure definitions (range: 5 to 43 infants across ages). However, infants meeting multiple definitions had a higher likelihood of developmental concerns outcomes at all ages. Positive predictive values for meeting all three measure definitions combined ranged from 0.67 [0.22, 0.96] at 6 months to 0.95 [0.75, 1.0] at 12 months. These findings suggest that while majority of infants ultimately classified with developmental concerns do not show significant behavioral differences in the first year, when early differences are present, they have predictive value. Combining behavioral tools may help to improve identification of infants most in need of early supports.

**Lay abstract:**

Many families have concerns about their infants’ development in the first year of life. Current screeners cannot tell whether these differences might be related to autism, developmental delays, or likely to resolve on their own. As a result, many families are told to “wait and see.” In this study, we looked at whether combining multiple behavior measures can improve prediction of outcomes in toddlerhood. This could help to provide families with more information about the significance of early behavioral differences. We assessed 256 infants with an older autistic sibling at 6, 9, and 12 months. We created three markers of behavioral differences at these ages. We looked at whether infants who had two or more markers were more likely to be on the autism spectrum or have other developmental differences than to have typically developing outcomes at 36 months. We found that very few infants had more than one marker at any age. However, infants who showed two or more markers were more likely to be on the spectrum or have other developmental differences at 36 months than infants who showed only one marker. These findings suggest that when behavioral differences are present on multiple measures, there is no need to wait and see before referring for services.

Many caregivers of autistic children report developmental concerns as early as the first or second years of life (Chawarska et al., 2007; Crane et al., 2016; De Giacomo & Fombonne, 1998; Herlihy et al., 2015). However, families face many barriers in accessing autism-specific evaluations and services at these young ages, including hesitancy by primary care providers to initiate referrals, recommendations to “wait and see,” long waitlists at centers with appropriate expertise, and limited numbers of community-based providers. This results in years between parents’ first concerns and their child’s ultimate diagnosis, and consequently, lost time to access supportive services during this important developmental period (Dababnah et al., 2018; Edwards et al., 2021; Zuckerman et al., 2015). One key challenge is the lack of tools available to guide clinical decision-making regarding an individual infant’s likelihood of autism or other non-typical outcomes during the first year. Development of such tools could help improve responsiveness to families with early developmental concerns, prioritize waitlists for specialty evaluations, and “fast track” infants most in need of early supportive services. In this article, we conducted a secondary data analysis of 256 infants with an older autistic sibling to examine whether combining multiple behavioral assessment tools could help identify individual infants with elevated likelihood of non-typical outcomes, including autism, in the first year of life.

There are several unique challenges in identifying infants with elevated likelihood for autism and other developmental differences in the first year of life. Most of the data on very early development in autism comes from studies of younger siblings of autistic children followed prospectively through toddlerhood when diagnostic outcomes can be ascertained. These studies have demonstrated that symptoms emerge over time and at different rates across children; even with multiple serial developmental evaluations, only approximately 40% of infants ultimately diagnosed with autism meet criteria by 18 months of age, with many others not meeting formal diagnostic criteria until 24 or 36 months (Chawarska et al., 2014; Ozonoff et al., 2015; Szatmari et al., 2016). Given this heterogeneity in the unfolding of autism symptoms, it is not surprising that existing behavioral assessments appropriate for use in the first year of life have relatively modest sensitivity (i.e. ability to identify autism among those ultimately diagnosed) and specificity (i.e. ability to accurately identify cases without autism in the sample), as behavioral signs may not yet be measurable in many children. For example, at 12 months, the Autism Observation Scale for Infants (AOSI) demonstrated 0.52 sensitivity and 0.74 specificity in predicting 36-month autism outcomes within an infant sibling sample (Zwaigenbaum, Bryson, et al., 2021).

A second key finding to emerge from this literature is that infant siblings have an elevated likelihood of atypical developmental outcomes more broadly, with approximately 20% meeting criteria for autism and another 20%–30% exhibiting other developmental differences such as elevated attention-deficit hyperactivity disorder (ADHD) and anxiety symptoms, speech-language problems, and lower scores on adaptive functioning measures (Messinger et al., 2013; Shephard et al., 2017). Thus, assessments used in the first year of life that identify multiple types of early delays, including but not limited to autism, could help more children benefit from early intervention.

## Improving detection in the first year of life

Screening tools used in the second year of life in general settings such as well-baby visits prioritize sensitivity and specificity in order to identify as many affected children as possible while limiting referrals for children who do not need them (McCarty & Frye, 2020). This approach may be less helpful in the first year of life when signs of autism are rarely clear (Zwaigenbaum, Bryson, et al., 2021; Zwaigenbaum, Brian, et al., 2021), limiting sensitivity. Instead, the challenge is to determine whether any behavioral differences present are clinically meaningful and predictive of later challenges. Prioritizing positive predictive value (PPV) over sensitivity and specificity may better address the specific needs of this developmental period. PPV is defined as the proportion of individuals scoring above a given threshold who have the outcome of interest. Developing tools with high PPV will be useful for guiding feedback to families with DC about their infant’s likelihood of autism or other developmental differences.

PPV is heavily influenced by the base rate of the outcome within a given population. This means that even screeners with high sensitivity and specificity tend to have poor PPV in general population samples, since the base rate of autism is relatively low (approximately 3%; Maenner et al., 2023). Therefore, it may be useful to examine PPV in a population with a higher base rate of autism and other developmental delays, such as infant siblings of autistic children or children for whom concerns have already been raised by parents or practitioners. Such an approach is consistent with increasing calls for a stepped and iterative approach to early screening wherein infants are initially identified based on universal level 1 screening or history (e.g. autistic sibling, prematurity, genetic syndrome, caregiver concerns), and subsequently evaluated using a level 2 screening or assessment to differentiate between autism or other developmental challenges (Dow et al., 2020; Green et al., 2022; Hyman et al., 2020; Khowaja et al., 2018; Robins, 2020; Sturner et al., 2023; Talbott & Miller, 2020; Wieckowski et al., 2022). This two-stage approach has been shown to improve diagnostic accuracy beginning in the second year of life (Brewer et al., 2020; Khowaja et al., 2018; McCarty & Frye, 2020; Towle & Patrick, 2016). We propose that applying this multi-stage framework could help to identify some infants in the first year of life with high likelihood of non-typical outcomes.

## This study

This study investigated the potential for existing behavioral assessments, when used in the first year of life, to identify infants with elevated likelihood of autism and other developmental differences in toddlerhood. That is, are there some infants for whom early behavioral differences are predictive of subsequent non-typical developmental outcomes? To answer this question, we examined the PPV of three behavioral measures collected at 6, 9, and 12 months of age, individually and in combination, to predict non-typical developmental outcomes at age 3. For each measure, we defined cutoffs representing clear developmental differences in the first year of life (markers). We hypothesized that combining multiple measures would decrease the number of false positives and therefore increase the PPV, and thus the clinical utility, of such a screening approach. Given the relatively low overall prevalence of autism and other non-typical outcomes among general population samples, this article focused on infants with a family history of autism (FH). If combining multiple measures yields greater predictive utility within this sample, future studies could test the tools in general community samples.

## Methods

### Participants

This is a secondary analysis of data collected from three cohorts enrolled in longitudinal studies of infants with (*n* = 256) or without (*n* = 144) an FH; primary analyses focus on the group with an FH. Data from those without an FH were used to determine cutoffs on some measures, as detailed below. Inclusion criteria for the FH group was status as the younger sibling of an autistic child. Diagnosis of the sibling was confirmed by meeting autism criteria on both the Autism Diagnostic Observation Schedule (ADOS) and the Social Communication Questionnaire (SCQ). Exclusion criteria for the FH group included birth before 36 weeks gestation or known genetic diagnosis (e.g. Fragile X Syndrome). Participants were recruited through a university registry, birth-record mailings, social media, websites targeted to autism, word of mouth (parents referring other parents), and fliers posted in the community.

### Procedures

Data were collected at multiple time points across the first 3 years of life for each cohort (at 6, 9, 12, 18, and 24 months of age), and a diagnostic outcome visit at 36 months of age. Participants in this analyses included all infants with an FH who had at least one assessment visit at 6, 9, or 12 months of age and a final visit at 36 months. Participants were evaluated at all ages by examiners unaware of family history and the results of prior assessments. Examiners were all MA or PhD level, with multiple years of experience in autism evaluations, and supervised by a licensed clinical psychologist who verified all diagnostic outcomes. Of the 256 FH participants, 158 had data at all three infant time points (6, 9, and 12), 47 had visits at 6 and 12 months, 30 had visits at 9 and 12 months, and 21 had visits at 12 months only. Demographic information and clinical outcomes for all participants are shown in Table 1. All data were collected at UC Davis Health, with approval from the Institutional Review Board. Written informed consent was obtained from all parents or legal guardians.

**Table 1.** Participant demographics.

Demographics	FH infants (*n* = 256)
Infant sex (*n*, % male)	145, 56.6%
Infant race (*n*, %)	
American Indian or Alaskan Native	5, 2%
Asian	29, 11.3%
Black or African American	3, 1.2%
More than one race	52, 20.3%
Native Hawaiian or Other Pacific Islander	3, 1.2%
Unknown or not reported	12, 4.7%
White	152, 59.4%
Infant ethnicity (*n*, %)	
Hispanic or Latino	49, 19.1%
Not Hispanic or Latino	194, 75.8%
Unknown or not reported	13, 5.1%
Maternal education (*n*, %)	
High school/GED/Vocational	25, 9.8%
Some college^a^	53, 20.7%
College degree	75, 29.3%
Graduate degree^b^	67, 26.2%
Not reported	37, 14.5%
36-Month Outcome Classification	
Autism	56, 21.9%
Non-typically developing	42, 16.4%
Typically developing	158, 61.7%

FH: family history of autism; GED: General Educational Development.

a Includes courses toward college and/or associates or 2-year degree.

b Includes Masters and Doctorate.

### Measures

#### Autism observation scale for infants

This semi-structured behavioral observation measure was designed to measure behavioral constructs related to autism in infants 6- to 18-months of age (Bryson et al., 2008). A series of play interactions and systematic presses assess target behaviors including visual tracking and attention disengagement, coordination of eye gaze and action, imitation, affective responses, early social-communicative behaviors, behavioral reactivity, and sensory–motor development. It has excellent inter-rater reliability and fair-to-good test–retest reliability on standard laboratory-based administration (Bryson et al., 2008). AOSI total scores range from 0 to 38. At 12 months, a cut-off score of 7 results in a relative risk ratio of 1.58, with infants having scores at or above 7 more likely to have an autism outcome (Sacrey et al., 2018). AOSI total score was used as one of the three marker variables, described further in the behavioral markers definitions section below.

#### Mullen scales of early learning

This standardized developmental measure for children from birth to 68 months contains four cognitive subscales: Expressive Language, Receptive Language, Visual Reception, and Fine Motor (Mullen et al., 1995). Raw scores can be converted to T-scores and age equivalents. The original Mullen norming sample demonstrated good internal, test–retest, and interrater reliability (Mullen, 1995). The Mullen scales of early learning (MSEL) also shows good convergent validity with other standardized tests of cognitive functioning (Bishop et al., 2011). In this study, an Overall developmental quotient (DQ) was calculated by dividing the mean age-equivalent score for the four subtests by the child’s chronological age and multiplying by 100.

#### Parent concerns

At the end of each visit, examiners asked parents to describe any “ . . . current concerns about [their child’s] development or behavior at this time” and recorded responses verbatim (Ozonoff et al., 2009). All responses were coded into one of eight categories of concerns or no concerns, following the procedures detailed by Ozonoff and colleagues (2009). In previous use of this instrument, parents of infants who are later diagnosed with autism report significantly more concerns than parents of infants with other developmental delays or typically developing outcomes beginning at 12 months of age (Ozonoff et al., 2009). The current study used a dichotomous variable indicating the presence/absence of autism-related concerns (defined as parent concerns about speech/language/communication, social development, repetitive behavior, and/or unspecified general worries about autism).

#### Behavioral marker definitions in infancy

We generated cutoffs for each of the three measures collected at 6, 9, and 12 months. MSEL scores were dichotomized based on standard scores, with low MSEL scores defined as a DQ less than or equal to 85 (e.g. 1 standard deviation below the mean). Parent concerns were dichotomized as absence or presence of autism-related concerns (none versus one or more). High AOSI scores at 12 months were defined as 7 or above, as recommended by the measure’s developers (Sacrey et al., 2018). There are no recommended cutoffs for 6 or 9 months of age because the receiver operating characteristics (ROC) analyses used to develop the existing guidelines did not statistically differentiate between infants with and without autism outcomes until 12 months of age (Zwaigenbaum, Bryson, et al., 2021). We defined high AOSI scores as 1.5 standard deviations above the mean of our no family history (NFH) group collected as part of the larger infant sibling study. High AOSI in our sample was defined as ⩾ 13 at 6 months and ⩾ 10 at 9 months. Of note, at 12 months, the published and recommended cutoff of 7 was identical to the cutoff generated using the 1.5 SD above the NFH group definition.

Using these cutoffs, we determined whether a child demonstrated one, two, or three of the behavioral markers and examined the psychometric properties of each marker, individually and in combination, at each age. Mean scores and standard deviations for the three measures collected at 6, 9, and 12 months for the FH and NFH groups are presented in Table 2. At the group level, the FH group had a significantly greater number of parent concerns across all ages. At 6 months, the FH group also had significantly lower MSEL DQ scores. By 12 months, the FH group also had significantly higher AOSI total scores than the NFH group.

**Table 2.** Sample characteristics, by recruitment group.

Age	Measure	FH infants		NFH infants	
		*N*	Mean (SD)	*N*	Mean (SD)
6 Months	AOSI total score	133	9.44 (4.2)	68	8.57 (3.4)
	MSEL DQ	174	93.46 (13.2)	108	96.60 (12.6)*
	Autism-related parent concerns	182	0.59 (1.0)	109	0.07 (0.3)***
9 Months	AOSI total score	90	5.91 (3.2)	58	5.36 (3.1)
	MSEL DQ	93	95.40 (11.8)	58	95.51 (11.4)
	Autism-related parent concerns	94	0.79 (1.3)	59	0.17 (0.5)***
12 Months	AOSI total score	181	5.31 (4.0)	94	3.46 (2.6)***
	MSEL DQ	250	101.35 (14.2)	139	106.90 (10.6)***
	Autism-related parent concerns	250	0.84 (1.2)	138	0.26 (0.53)***

FH: family history of autism; NFH: non-FH; AOSI: Autism Observation Scale for Infants; MSEL: Mullen Scales of Early Learning; DQ: developmental quotient.

* *p* *<* 0.05, **p < 0.01, ***p < 0.001.

### 36-Month diagnostic outcome measures and procedures

#### Autism diagnostic observation schedule (ADOS), second edition

This is a semi-structured, play-based interaction and observation tool assessing social communication and repetitive behavior (Lord et al., 2012). Comparison scores, ranging from 1 to 10, allow for comparison of ADOS scores across different administration modules, with scores of 4 and above indicative of autism (Gotham et al., 2009). Here, the ADOS was used to confirm older sibling diagnosis and to determine 36-month infant outcomes.

#### Outcome classification procedure

At 36 months, infants were classified into three outcome groups: autism, typical development (TD), or non-typical development (Non-TD), following the procedures of Ozonoff and colleagues (2014). Criteria for the autism outcome group included (1) comparison score at or above the autism spectrum disorder (ASD) cutoff of 4 on the ADOS-2 and (2) meeting Diagnostic and Statistical Manual of Mental Disorders (5th ed.; *DSM* V) criteria for ASD. Criteria for the TD outcome group included (1) not meeting autism outcome criteria, (2) ADOS-2 comparison score of 2 or below, (3) no more than 1 MSEL subscale score ⩾ 1.5 SD below the mean, and (4) no MSEL subscale score ⩾ 2 SD below the mean. Criteria for the Non-TD outcome included (1) does not meet autism outcome criteria, (2) Two or more MSEL subscale scores ⩾ 1.5 SD below the mean, and/or (3) one or more MSEL subscale scores ⩾ 2 SD below the mean, and/or (4) ADOS-2 comparison score of 3 or higher. Based on this algorithmic definition, 56 infants met criteria for the autism outcome, 42 for Non-TD, and 158 for TD. For the purposes of this analyses, the autism and Non-TD groups were combined into a single developmental concerns (DC) group.

Community involvement statement: community members were not involved in this study.

## Results

Mean scores and standard deviations for the three measures collected at 6, 9, and 12 months for the TD and DC groups are presented in Table 3. At the group level, the DC group had significantly higher AOSI scores than the TD outcome group by 9 months, and a significantly greater number of parent concerns and lower MSEL DQ scores at 12 months.

**Table 3.** Sample characteristics, by outcome group, FH group only.

Age	Measure	TD outcome		DC outcome	
		*N*	Mean (SD)	*N*	Mean (SD)
6 Months	AOSI total score	77	9.09 (4.2)	56	9.93 (4.2)
	MSEL DQ	110	94.94 (12.8)	64	90.92 (13.5)
	Autism-related parent concerns	111	0.59 (1.1)	71	0.61 (0.92)
9 Months	AOSI total score	54	5.39 (2.5)	36	6.69 (3.9)**
	MSEL DQ	56	98.29 (11.1)	37	91.03 (11.7)
	Autism-related parent concerns	57	0.79 (1.4)	37	0.79 (1.1)
12 Months	AOSI total score	107	4.40 (3.1)	74	6.64 (4.47)***
	MSEL DQ	155	105.51 (12.4)	95	94.56 (14.4)***
	Autism-related parent concerns	155	0.71 (1.0)	95	1.1 (1.5)*

FH: family history of autism; TD: typical development; DC: developmental concerns; AOSI: Autism Observation Scale for Infants; MSEL: Mullen Scales of Early Learning; DQ: developmental quotient.

* *p* < 0.05, **p < 0.01, ***p < 0.001.

We started by examining whether there were fewer infants who met the multiple marker versus single marker definitions. Across all ages and outcome groups, few infants demonstrated all three behavioral markers (only 6 at 6 months, 5 at 9 months, and 13 at 12 months). The number of infants with two behavioral markers was higher, though still a small number of infants overall. Table 4 lists the distribution of infants at or above the cutoffs on single and combined marker definitions at each of the three ages.

**Table 4.** Number of infants scoring above each marker definition, by age, FH group only.

Age	Definition	TD outcome			DC outcome		
		Total	Marker present	Marker absent	Total	Marker present	Marker absent
6 Months	Any 1	112	61	51	71	45	26
	AOSI	77	15	62	56	15	41
	MSEL	110	24	86	64	21	43
	PC	111	38	73	71	30	41
	AOSI + PC	46	4	72	56	7	49
	AOSI + MSEL	77	7	70	53	9	44
	MSEL + PC	109	7	102	64	9	55
	Any 2	110	14	96	67	17	50
	All 3	76	2	74	53	4	49
9 Months	Any 1	57	28	29	37	26	11
	AOSI	54	4	50	36	8	48
	MSEL	56	8	48	37	11	26
	PC	57	22	35	37	19	18
	AOSI + PC	54	3	51	36	5	31
	AOSI + MSEL	54	1	53	36	5	31
	MSEL + PC	56	3	53	37	6	31
	Any 2	56	5	51	37	8	29
	All 3	54	1	53	36	4	32
12 Months	Any 1	155	81	74	95	66	29
	AOSI	107	22	85	74	35	39
	MSEL	155	6	149	95	27	68
	PC	155	66	89	95	47	48
	AOSI + PC	107	10	97	74	18	56
	AOSI + MSEL	107	1	106	74	19	55
	MSEL + PC	155	3	152	95	18	77
	Any 2	155	12	143	95	31	64
	All 3	107	1	106	74	12	62

FH: family history of autism; TD: typical development; DC: developmental concerns; AOSI: Autism Observation Scale for Infants; MSEL: Mullen Scales of Early Learning; PC: autism-related parent concerns.

The Any 1, Any 2, or All 3 definitions includes infants whose scores met behavior marker definitions on at least one, at least two, or all three of the measures.

Differences in the relative proportions of infants meeting the single versus multiple marker definitions were explored using McNemar’s exact tests, comparing the proportion of infants who met any single-marker definition (“Any 1”) versus the Any 2 marker definition in infants for whom at least two measures were available at a given age. Across all three ages, the proportions of infants who met the Any 2 definition were significantly smaller than the Any 1 definition (all *p* < 0.001). Thus, as expected, fewer infants met the multiple measure definitions as compared to individual measure cutoffs, across all ages. This suggests that combining multiple markers may help to reduce the number of false positives, or infants with typically developing outcomes who have transiently elevated scores on one single measure. For example, at 6 months, more than half the infants with typically developing outcomes scored above the cutoff on at least one measure (61 of 122; 54%), but only 14 (of 110; 13%) scored above the cutoff on two measures.

Next, we examined how well each definition performed in identifying infants belonging to the typically developing and DC groups at 36 months. We calculated the PPV, negative predictive value (NPV), sensitivity, and specificity of each definition in predicting classification into the DC group at 36 months (see Table 5). At each age, the behavioral marker definition with the highest PPV involved the combination of at least two measures. At 6 months, this was the All 3 definition, with a PPV of 0.67 (95% confidence interval (CI): 0.22–0.96). At 9 and 12 months, PPV was highest for the AOSI + MSEL definition, 0.83 (95% CI: 0.36–1.0) and 0.95 (95% CI: 0.75–1.0), respectively.

**Table 5.** PPV, NPV, sensitivity, and specificity of marker definitions, by age, for DC outcomes versus TD outcomes, FH group only.

Age	Definition	Positive predictive value (PPV) [95% CI]	Negative predictive value (NPV) [95% CI]	Sensitivity [95% CI]	Specificity [95% CI]
6 Months	Any 1	0.42 [0.32–0.52]	0.66 [0.55–0.77]	0.63 [0.51–0.75]	0.46 [0.36–0.55]
	AOSI	0.50 [0.31–0.69]	0.60 [0.50–0.70]	0.27 [0.16–0.40]	0.81 [0.70–0.88]
	MSEL	0.47 [0.31–0.62]	0.67 [0.58–0.75]	0.33 [0.22–0.46]	0.78 [0.70–0.86]
	PC	0.44 [0.32–0.57]	0.64 [0.55–0.73]	0.42 [0.31–0.55]	0.66 [0.56–0.75]
	AOSI + PC	0.64 [0.31–0.89]	0.60 [0.50–0.68]	0.13 [0.05–0.24]	0.95 [0.87–0.99]
	AOSI + MSEL	0.56 [0.30–0.80]	0.61 [0.52–0.70]	0.17 [0.08–0.30]	0.91 [0.82–0.96]
	MSEL + PC	0.56 [0.30–0.80]	0.65 [0.57–0.73]	0.14 [0.07–0.25]	0.94 [0.87–0.97]
	Any 2	0.55 [0.36–0.72]	0.66 [0.58–0.73]	0.25 [0.16–0.38]	0.87 [0.80–0.93]
	All 3	0.67 [0.22–0.96]	0.60 [0.51–0.69]	0.08 [0.02–0.18]	0.97 [0.91–1.0]
9 Months	Any 1	0.48 [0.34–0.62]	0.73 [0.56–0.85]	0.70 [0.53–0.84]	0.51 [0.37–0.64]
	AOSI	0.67 [0.35–0.90]	0.51 [0.52–0.75]	0.14 [0.10–0.39]	0.93 [0.82–0.98]
	MSEL	0.58 [0.34–0.80]	0.65 [0.53–0.76]	0.30 [0.16–0.47]	0.86 [0.74–0.94]
	PC	0.46 [0.31–0.63]	0.66 [0.51–0.73]	0.51 [0.34–0.68]	0.61 [0.48–0.74]
	AOSI + PC	0.63 [0.25–0.92]	0.62 [0.51–0.73]	0.14 [0.05–0.30]	0.94 [0.85–0.99]
	AOSI + MSEL	0.83 [0.36–1.0]	0.63 [0.52–0.73]	0.14 [0.05–0.30]	0.98 [0.90–1.0]
	MSEL + PC	0.67 [0.30–0.93]	0.63 [0.52–0.73]	0.16 [0.07–0.25]	0.95 [0.85–0.99]
	Any 2	0.62 [0.31–0.86]	0.64 [0.52–0.74]	0.22 [0.10–0.38]	0.91 [0.80–0.97]
	All 3	0.80 [0.28–1.0]	0.62 [0.51–0.73]	0.11 [0.03–0.26]	0.98 [0.90–1.0]
12 Months	Any 1	0.44 [0.37–0.53]	0.72 [0.62–0.80]	0.69 [0.59–0.79]	0.48 [0.40–0.56]
	AOSI	0.61 [0.48–0.74]	0.69 [0.55–0.77]	0.47 [0.36–0.59]	0.79 [0.71–0.87]
	MSEL	0.81 [0.65–0.93]	0.69 [0.62–0.75]	0.28 [0.20–0.39]	0.96 [0.92–0.99]
	PC	0.41 [0.32–0.51]	0.65 [0.55–0.71]	0.49 [0.39–0.60]	0.57 [0.49–0.65]
	AOSI + PC	0.64 [0.44–0.81]	0.63 [0.55–0.71]	0.24 [0.15–0.36]	0.91 [0.85–0.95]
	AOSI + MSEL	0.95 [0.75–1.0]	0.66 [0.58–0.73]	0.26 [0.16–0.37]	0.99 [0.95–1.0]
	MSEL + PC	0.86 [0.64–0.97]	0.66 [0.60–0.73]	0.19 [0.12–0.28]	0.98 [0.94–1.0]
	Any 2	0.72 [0.56–0.85]	0.69 [0.62–0.75]	0.33 [0.23–0.43]	0.92 [0.87–0.96]
	All 3	0.93 [0.64–1.0]	0.63 [0.55–0.70]	0.16 [0.09–0.27]	0.99 [0.95–1.0]

DC: developmental concerns; TD: typical development; FH: family history of autism; CI: confidence interval; AOSI: Autism Observation Scale for Infants; MSEL: Mullen Scales of Early Learning; PC: autism-related parent concerns.

The Any 1, Any 2, or All 3 definitions includes infants whose scores met behavior marker definitions on at least one, at least two, or all three of the measures.

The high PPV for these combined marker definitions contrasts with their low sensitivity values, which were expected and consistent with previous research showing most infants ultimately classified with DC do not demonstrate clear differences in the first year of life (Cleary et al., 2023; Zwaigenbaum et al., 2015). However, the high PPVs suggest that when multiple developmental differences are present, there is a strong likelihood those differences will persist into toddlerhood. At both 9 and 12 months, all but one infant who met either the AOSI + MSEL or All 3 definitions were classified into the DC group at outcome. Furthermore, of the 13 infants who met the All 3 definition at 12 months, 11 were diagnosed with ASD, 1 classified as Non-TD, and only 1 was typically developing. Supplemental Table 1 shows the number of infants who scored above each marker definition separately for autism and Non-TD outcomes. Supplemental Table 2 presents the associated PPV, NPV, sensitivity, and specificity values for differentiating autism from Non-TD outcomes. In general, the pattern of findings was similar in the autism-only outcome group, with the highest PPV at each age involving the combination of at least two measures. As expected, given the smaller sample size in this sub-group, the 95% CIs were wider and overlapped across all definitions. Sensitivity was also quite low across most definitions.

**Figure 1.**
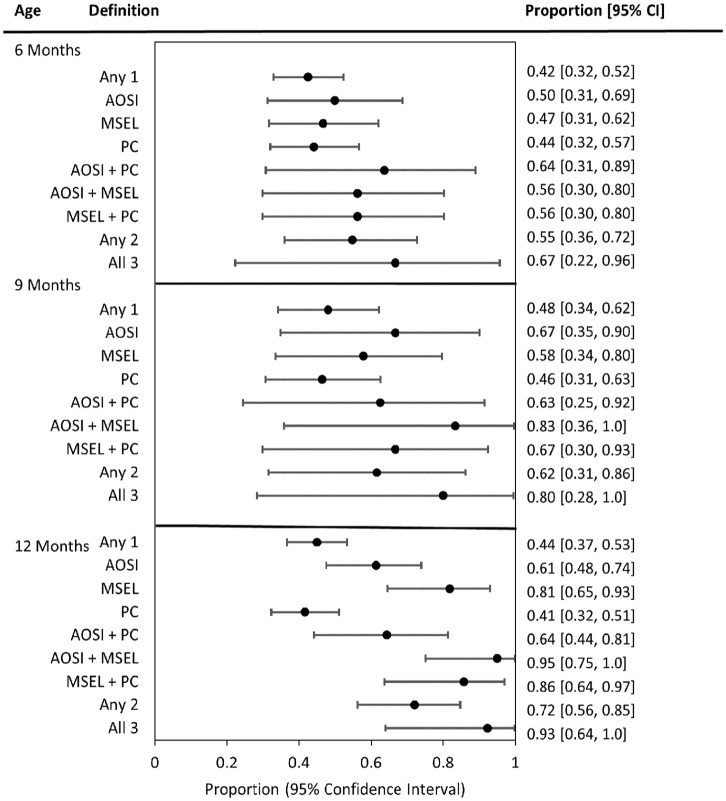
PPV with confidence intervals for DC outcomes, by age and marker definition, FH group only. AOSI: Autism Observation Scale for Infants; MSEL: Mullen Scales of Early Learning; PC: autism-related parent concerns. The Any 1, Any 2, or All 3 definitions includes infants whose scores met behavior marker definitions on at least one, at least two, or all three of the measures.

Because the measures available at each age differed across cohorts and because the criteria for each marker definition were not independent, it was not possible to directly compare each definition pair. Instead, we examined differences in the PPV values for the single-marker (e.g. AOSI, MSEL, PC, and Any 1) versus multiple marker (e.g. AOSI + PC, AOSI + MSEL, MSEL + PC, Any 2, All 3) definitions by calculating the 95% CI for each definition at each age. Non-overlapping CIs suggest a true difference in the proportion of infants scoring above the cutoff who belonged to the DC outcome group. These estimates are included in Table 5, and can be visualized in Figure 1 to facilitate comparison across the different definitions.

As can be seen in Figure 1, although the PPVs for the multiple marker definitions were generally higher than the single-marker definitions across all ages, at both 6 and 9 months, the 95% CIs for these proportion estimates overlapped across all definitions. This suggests that the multiple-marker definitions may not identify a significantly higher proportion of infants with DC outcomes than the single-marker definitions at these earliest ages. However, at 12 months, the proportion of infants with DC correctly identified by the MSEL alone, AOSI + MSEL, MSEL + PC, Any 2, and All 3 definitions were all significantly higher than Any 1 or Parent Concerns alone, as these CIs do not overlap. The AOSI + MSEL definition also identified a significantly higher proportion of DC outcomes infants than the AOSI alone, as the 95% CIs do not overlap. Importantly, 19 of 20 infants meeting the AOSI + MSEL definition belonged to the DC outcomes group. This suggests that when infants are showing a combination of high AOSI and low MSEL scores, there is a high likelihood these differences will persist into toddlerhood and result in a classification of autism or other Non-TD.

**Figure 2.**
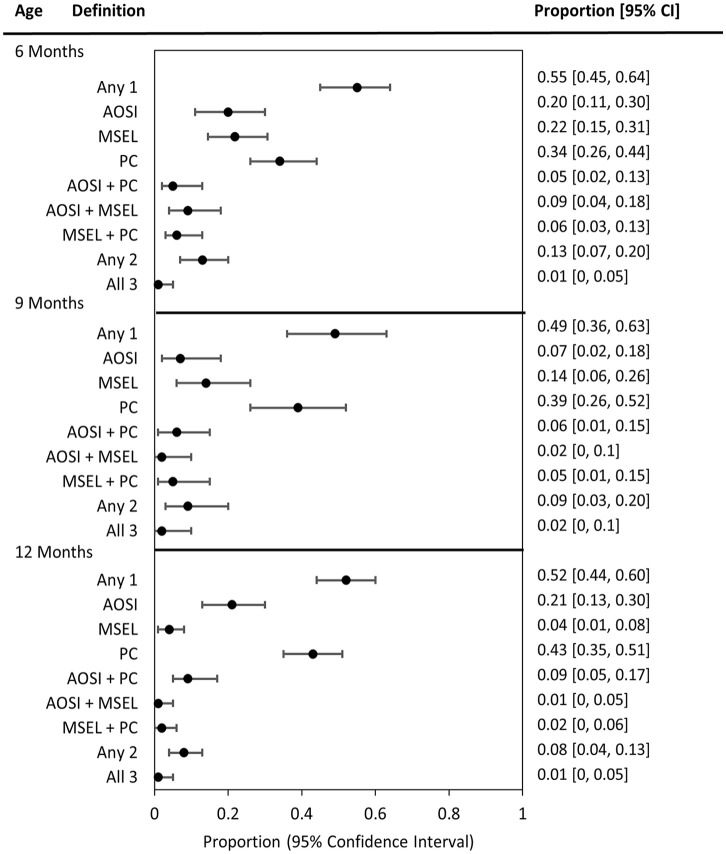
PPV with confidence intervals for TD outcomes, by age and marker definition, FH group only. AOSI: Autism Observation Scale for Infants; MSEL: Mullen Scales of Early Learning; PC: autism-related parent concerns. The Any 1, Any 2, or All 3 definitions includes infants whose scores met behavior marker definitions on at least one, at least two, or all three of the measures.

We used the same CI approach to compare differences in the proportion of infants with TD outcomes who met the single-marker (e.g. AOSI alone) versus multiple marker (e.g. AOSI + MSEL) definitions. This represents the proportion of infants incorrectly flagged by the each of the definitions (e.g. the false-positive rate or FPR). As can be seen in Figure 2, across all ages, the multiple-marker definitions resulted in proportionally fewer infants incorrectly identified. At all ages, the CIs around the estimates for the Any 1 and Parent Concerns Alone were higher than, and did not overlap with, any of the multiple-marker definitions. This indicates these two measures incorrectly identified a significantly higher proportion of infants with TD outcomes than the other definitions. At 6 and 9 months, the intervals around the AOSI and MSEL alone and each of the multiple-marker definitions all overlapped, suggesting that although a smaller proportion of infants were incorrectly identified by the multiple-marker definitions than the AOSI or MSEL alone, the differences were not significant. At 12 months, the MSEL alone, AOSI + MSEL, MSEL + PC, and All 3 definitions identified a significantly smaller proportion of infants with typical outcome classifications than the Any 1, AOSI alone, and Parent Concerns definitions. Thus, a similar pattern of improved identification rates was observed in the FPR as the PPV, with the multiple-marker definitions associated with fewer infants falsely identified than the AOSI alone, particularly at 12 months.

Overall, we found that combining multiple behavioral markers increased the proportion of infants scoring above the cutoff who are ultimately classified into the DC group (e.g. PPV), particularly at 12 months. At 6 months, the highest PPV was observed for the high AOSI and parent concern definition (PPV 0.64); adding low Mullen scores increases PPV slightly (0.67). While the combined marker definitions had higher PPV than the single-measure marker definitions, the 95% CIs for each proportion estimate overlaps for all definitions. This suggests that at 6 months, combining multiple markers does not significantly improve PPV. At 9 months, the highest PPV is obtained for the definition involving high AOSI and low MSEL scores (PPV 0.83). However, the 95% CIs around the proportion estimates overlap for all definitions, again suggesting that at 9 months, combining multiple markers does not significantly improve PPV. At 12 months, the highest PPV is again observed for the high AOSI and low MSEL definition (PPV 0.95). This definition significantly improves PPV over AOSI alone, parent concerns, or Any 1 definitions. Nineteen of 20 infants with both high AOSI and low MSEL scores at 12 months were classified into the DC outcome group.

## Discussion

In this study, we examined the potential of combining three early behavioral measures to identify infants 6–12 months with elevated likelihood of DC, including autism or other non-typical outcomes, in toddlerhood. We generated cutoffs for each of three measures collected at 6, 9, and 12 months: the AOSI, Mullen Scales of Early Learning (MSEL), and the presence of autism-related Parent Concerns (PC). We found that combining two or more measures yielded a PPV between 0.67 and 0.95 in predicting any non-typical outcome (including autism as well as other DC, such as speech-language delays). While many infants with typical development outcomes met single-marker definitions, very few met multiple marker definitions. For example, at 6 months, 15 infants with TD outcomes met the high AOSI score definition, but only 7 met both the high AOSI and low MSEL definition. At 12 months, 22 infants with TD outcomes met the high AOSI definition, yet only one participant with a typical outcome at 36 months met both high AOSI and low MSEL markers. This suggests that using this multiple-marker approach could reduce the number of infants unnecessarily referred for additional assessments or services.

As expected, sensitivity was low across all ages and groups. This is consistent with the existing literature demonstrating the heterogeneous developmental patterns among infant siblings of autistic children and the protracted emergence of developmental differences in toddlerhood. It is clear that repeated serial screening will be needed to progressively identify infants and toddlers over time (Lipkin, 2023). Our findings build on this literature by identifying one potential approach to identifying DC at the very earliest ages, requiring multiple behavioral differences across multiple domains. While differentiating between autism and other developmental differences is, by definition, not possible during the pre-diagnostic period, we suggest that infants demonstrating differences on multiple measures are those most likely to benefit from referrals and services. Existing autism screeners for older toddlers also perform much better when predicting developmental differences that are more broadly defined, rather than autism specifically. For example, the initial validation of the M-CHAT-R/F reported a PPV of 0.48 (95% CI: 0.41–0.54) in predicting autism specifically; it rose to 0.95 (95% CI: 0.92–0.98) when predicting any developmental condition (e.g. Global Developmental Delay, Language Disorder). For this reason, we used a broader outcome classification that included not only autism but also other developmental delays and/or clinical concerns, as defined by elevated scores on the ADOS-2 and/or low scores on the Mullen. These infants are likely to benefit from evaluation and supports, regardless of specific diagnostic outcome. It is worth noting that we observed a similar pattern of results, with similar PPV and sensitivity indices, when predicting to autism-specific outcomes as well, further demonstrating the utility of this approach.

### Clinical Implications

Although our sample is small, we suggest our approach offers a new lens for thinking about early detection processes. Existing recommendations and detection practices are inconsistent. For example, the US Preventative Services Task Force Recommendation Statement found insufficient evidence to assess the balance of benefits and harms for autism screening (Siu, 2016). However, the American Academy of Pediatrics (AAP) recommends integration of autism-specific screening at 18 and 24 months embedded within ongoing developmental surveillance through routine primary care visits (Hyman et al., 2020; Lipkin & Macias, 2020). There have now been several trials demonstrating the effectiveness of this approach in improving screening and autism identification rates in community settings (Barbaro et al., 2022; Karlov et al., 2024; McNally Keehn et al., 2020). However, a key challenge is the lack of tools validated for screening infants in the first year of life. The most recent AAP guidance lists several screening tools under development for infants less than 18 months of age (Hyman et al., 2020). However, only one of these includes infants younger than 12 months: The Infant/Toddler Checklist (ITC) from the Communication and Symbolic Behavior Scales (Wetherby et al., 2008). Prior work with the ITC in a sample of infant siblings of autistic children reported PPV ranging from 31% at 6 months to 62% at 24 months in predicting outcomes of any developmental delay (Autism + Non-TD; Parikh et al., 2021). In light of these challenges, there have been increased calls for repeated early screening and successive identification of infants most likely to benefit from referrals for evaluations and services at the time of screening (Constantino et al., 2021; Dai et al., 2020; Eisenhower et al., 2021; Robins, 2020).

Our findings are consistent with this serial screening and evaluation approach. Rather than trying to identify all infants who will ultimately be classified in the DC group (i.e. prioritizing sensitivity), we focused on identifying infants with the highest likelihood for autism and other developmental differences at each timepoint (i.e. prioritizing PPV). We suggest that for infants showing developmental differences across multiple domains, there is no need to “wait and see” to initiate services. When parents or others have concerns about infants’ development, they should be referred for developmental evaluation, and if clear behavioral differences are observed on multiple tools, they should be connected with early intervention services. Currently, these services might include existing low-intensity early intervention programs such as those administered through federally funded programs in the United States (Individuals with Disabilities Education Improvement Act, 2004). Our findings also support the development of additional transdiagnostic supports for infants and their families during this pre-diagnostic period (Charman et al., 2023; Constantino et al., 2021; Finlay-Jones et al., 2019). These supports might also include heightened developmental surveillance, including more frequent or repeated screening and evaluation of social communication development.

### Limitations and Future Directions

There are some limitations to this approach. First is the small number of infants who met multiple screening criteria and dependencies in the multiple marker definitions, which limits statistical power to compare across definitions and ages. This limitation is inherent to comparisons of PPV values, since PPV is calculated as a proportion of the number of infants who met the screening criteria for each definition. This means the denominator varies across PPV definitions, even within a single age, prohibiting the direct comparison of PPV values within a single set of participants. This is not an issue when comparing sensitivity values, in which the denominator (i.e. the total number of children with the outcome of interest) remains constant and only the numerator (i.e. the number of children who meet the screening criteria for each definition) varies. A second related limitation of this study is that infants were from families already familiar with autism, since they had an older diagnosed child. Such infants, and their families, may not be representative of community-ascertained samples. The use of standardized behavioral measures administered by well-trained examiners is also much more costly to implement than brief screening questionnaires so the methods described here may not generalize to clinical settings. However, the goal was more narrowly focused on understanding the clinical significance of early behavioral differences, rather than testing any specific screening tool. An important strength of the infant sibling design used here is the ability to follow the entire cohort of children—including those who screen negative—which is often not feasible in large community-based screening studies. While costly to implement, the use of standardized behavioral assessments supports the validity of our findings. There is also some initial evidence that the AOSI performs similarly in community-ascertained infants as in infant sibling samples (Hudry et al., 2021; Talbott et al., 2022). Future work focused on longitudinal examination of infants ascertained on the basis of DC from parents, providers, and/or universal screening tools, which will help to clarify the utility of this approach for identifying infants with the highest likelihood of non-typical outcomes. In particular, it will be helpful to examine the impact of combining multiple tools within the context of a developmental surveillance approach.

## Conclusion

This study demonstrates an approach that can identify infants with an elevated likelihood of autism and other non-typical outcomes in the first year of life. Further refinement of this approach may help to improve early detection practices by identifying which infants to prioritize for referrals. Our findings underscore the need for continued development of strategies for repeated screening and developmental monitoring. Overall, our findings indicate that although only a small number of infants will be identified at these very young ages, when significant developmental differences are present, particularly differences across multiple measures, they are likely to be clinically meaningful.

## Supplemental Material

sj-docx-1-aut-10.1177_13623613241275455 – Supplemental material for Can combining existing behavioral tools improve identification of infants at elevated likelihood of autism in the first year of life? Supplemental material, sj-docx-1-aut-10.1177_13623613241275455 for Can combining existing behavioral tools improve identification of infants at elevated likelihood of autism in the first year of life? by Meagan R Talbott, Gregory S Young and Sally Ozonoff in Autism
