# Supplementary material for: Can combining existing behavioral tools improve identification of infants at elevated likelihood of autism in the first year of life?
Source: Autism. 2024 Sep 12;29(2):462–75. doi: 10.1177/13623613241275455 (PMC11816472; doi:10.1177/13623613241275455)
Supplement: sj-docx-1-aut-10.1177_13623613241275455 – Supplemental material for Can combining existing behavioral tools improve identification of infants at elevated likelihood of autism in the first year of life? [file sj-docx-1-aut-10.1177_13623613241275455.docx]

**Supplemental Table 1.**

*Number of Infants Scoring Above Each Marker Definition, by Age, FH Group Only, Autism Versus Non-TD Outcomes*

| Age | Definition | Non-TD Outcome | | | Autism Outcome | | |
| --- | --- | --- | --- | --- | --- | --- | --- |
|  |  | **Total** | **Marker Present** | **Marker Absent** | **Total** | **Marker Present** | **Marker Absent** |
| 6 Months | **Any 1** | 29 | 19 | 10 | 42 | 26 | 16 |
|  | **AOSI** | 21 | 5 | 14 | 35 | 10 | 25 |
|  | **MSEL** | 28 | 11 | 17 | 36 | 10 | 26 |
|  | **PC** | 29 | 12 | 17 | 42 | 18 | 24 |
|  | **AOSI + PC** | 21 | 3 | 18 | 35 | 4 | 31 |
|  | **AOSI + MSEL** | 21 | 3 | 18 | 32 | 6 | 26 |
|  | **MSEL + PC** | 28 | 4 | 24 | 36 | 5 | 31 |
|  | **Any 2** | 28 | 8 | 20 | 39 | 9 | 30 |
|  | **All 3** | 20 | 1 | 20 | 32 | 3 | 29 |
| 9 Months | **Any 1** | 17 | 12 | 5 | 20 | 14 | 6 |
|  | **AOSI** | 17 | 3 | 14 | 19 | 5 | 14 |
|  | **MSEL** | 17 | 6 | 11 | 20 | 5 | 15 |
|  | **PC** | 17 | 6 | 11 | 20 | 13 | 7 |
|  | **AOSI + PC** | 17 | 1 | 16 | 19 | 4 | 15 |
|  | **AOSI + MSEL** | 17 | 2 | 15 | 19 | 3 | 16 |
|  | **MSEL + PC** | 17 | 1 | 16 | 20 | 5 | 15 |
|  | **Any 2** | 17 | 2 | 15 | 20 | 6 | 14 |
|  | **All 3** | 17 | 1 | 16 | 19 | 3 | 16 |
| 12 Months | **Any 1** | 41 | 25 | 16 | 54 | 41 | 13 |
|  | **AOSI** | 30 | 11 | 19 | 44 | 24 | 20 |
|  | **MSEL** | 41 | 7 | 34 | 54 | 20 | 34 |
|  | **PC** | 41 | 16 | 25 | 54 | 31 | 23 |
|  | **AOSI + PC** | 30 | 2 | 28 | 11 | 16 | 28 |
|  | **AOSI + MSEL** | 30 | 6 | 54 | 44 | 13 | 31 |
|  | **MSEL + PC** | 41 | 2 | 39 | 54 | 16 | 38 |
|  | **Any 2** | 41 | 8 | 33 | 54 | 23 | 31 |
|  | **All 3** | 30 | 1 | 29 | 44 | 11 | 33 |

*Note.* AOSI - Autism Observation Scale for Infants, MSEL – Mullen Scales of Early Learning, PC – Autism-Related Parent Concerns. The Any 1, Any 2, or All 3 definitions includes infants whose scores met behavior marker definitions on at least one, at least two, or all three of the measures.

**Supplemental Table 2.**

*PPV, NPV, Sensitivity, and Specificity of Marker Definitions, by Age, for Autism Outcomes versus Non-TD outcomes, FH Group Only*

| Age | Definition | Positive Predictive Value (PPV)[95%CI] | Negative Predictive Value (NPV) [95%CI] | Sensitivity [95%CI] | Specificity [95%CI] |
| --- | --- | --- | --- | --- | --- |
| 6 Months | **Any 1** | 0.58 [0.42 – 0.72] | 0.39 [0.20 – 0.59] | 0.62 [0.46 – 0.76] | 0.35 [0.18 – 0.54] |
|  | **AOSI** | 0.67 [0.38 – 0.88] | 0.39 [0.24 – 0.56] | 0.29 [0.15 – 0.46] | 0.76 [0.53 – 0.92] |
|  | **MSEL** | 0.48 [0.26 – 0.70] | 0.40 [0.25 – 0.56] | 0.28 [0.14 – 0.45] | 0.61 [0.41 – 0.79] |
|  | **PC** | 0.60 [0.41 – 0.77] | 0.42 [0.26 – 0.58] | 0.43 [0.28 – 0.59] | 0.59 [0.39 – 0.77] |
|  | **AOSI + PC** | 0.57 [0.18 – 0.90] | 0.37 [0.23 – 0.52] | 0.11 [0.03 – 0.27] | 0.86 [0.64 – 0.97] |
|  | **AOSI + MSEL** | 0.67 [0.30 – 0.93] | 0.41 [0.26 – 0.57] | 0.18 [0.07 – 0.36] | 0.86 [0.67 – 0.96] |
|  | **MSEL + PC** | 0.56 [0.21 – 0.86] | 0.44 [0.30 – 0.58] | 0.14 [0.05 – 0.30] | 0.86 [0.67 – 0.96] |
|  | **Any 2** | 0.53 [0.28 – 0.77] | 0.40 [0.26 – 0.55] | 0.23 [0.11 – 0.39] | 0.71 [0.51 – 0.87] |
|  | **All 3** | 0.75 [0.19 – 0.99] | 0.41 [0.27 – 0.56] | 0.09 [0.02 – 0.25] | 0.95 [0.76 – 1.0] |
| 9 Months | **Any 1** | 0.54 [0.33 – 0.73] | 0.46 [0.17 – 0.77] | 0.70 [0.46 – 0.88] | 0.29 [0.10 – 0.56] |
|  | **AOSI** | 0.63 [0.25 – 0.92] | 0.50 [0.31 – 0.69] | 0.26 [0.09 – 0.51] | 0.82 [0.57 – 0.96] |
|  | **MSEL** | 0.46 [0.17 – 0.77] | 0.42 [0.23 – 0.63] | 0.25 [0.09 – 0.49] | 0.65 [ 0.38 – 0.86] |
|  | **PC** | 0.68 [0.43 – 0.87] | 0.62 [0.36 – 0.83] | 0.65 [0.41 – 0.85] | 0.65 [0.38 – 0.86] |
|  | **AOSI + PC** | 0.80 [0.28 – 1.0] | 0.52 [0.33 – 0.64] | 0.21 [0.06 – 0.46] | 0.94 [0.71 – 1.0] |
|  | **AOSI + MSEL** | 0.60 [0.15 – 0.95] | 0.48 [0.30 – 0.67] | 0.16 [0.03 – 0.39] | 0.88 [0.64 – 0.99] |
|  | **MSEL + PC** | 0.83 [0.36 – 1.0] | 0.52 [0.33 – 0.70] | 0.25 [0.09 – 0.49] | 0.94 [0.71 – 1.0] |
|  | **Any 2** | 0.75 [0.35 – 0.97] | 0.52 [0.33 – 0.71] | 0.30 [0.11 – 0.39] | 0.88 [0.64 – 0.99] |
|  | **All 3** | 0.75 [0.19 – 0.99] | 0.50 [0.32 – 0.68] | 0.16 [0.03-0.40] | 0.94 [0.71 – 1.0] |
| 12 Months | **Any 1** | 0.62 [0.49 – 0.74] | 0.55 [0.36 – 0.74] | 0.76 [0.62 – 0.87] | 0.39 [0.24 – 0.56] |
|  | **AOSI** | 0.69 [0.51 – 0.83] | 0.49 [0.32 – 0.65] | 0.55 [0.39 – 0.70] | 0.63 [0.44 – 0.80] |
|  | **MSEL** | 0.74 [0.54 – 0.89] | 0.50 [0.38 – 0.62] | 0.37 [0.24 – 0.51] | 0.83 [0.68 – 0.93] |
|  | **PC** | 0.66 [0.51 – 0.79] | 0.52 [0.33 – 0.70] | 0.57 [0.43 – 0.71] | 0.61 [0.45 – 0.76] |
|  | **AOSI + PC** | 0.68 [0.43 – 0.87] | 0.50 [0.36 – 0.64] | 0.36 [0.22 – 0.52] | 0.93 [0.78 – 0.99] |
|  | **AOSI + MSEL** | 0.68 [0.43 – 0.87] | 0.44 [0.30 – 0.58] | 0.30 [0.17 – 0.45] | 0.80 [0.61 – 0.92] |
|  | **MSEL + PC** | 0.89 [0.65 -0.97] | 0.51 [0.39 – 0.62] | 0.30 [0.18 – 0.44] | 0.95 [0.84 – 0.99] |
|  | **Any 2** | 0.74 [0.55 – 0.88] | 0.52 [0.39 – 0.64] | 0.43 [0.29 – 0.57] | 0.88 [0.65 – 0.91] |
|  | **All 3** | 0.92 [0.62 – 1.0] | 0.47 [0.34 – 0.60] | 0.25 [0.13 – 0.40] | 0.97 [0.83 – 1.0] |
|  |  |  |  |  |  |

*Note.* AOSI - Autism Observation Scale for Infants, MSEL – Mullen Scales of Early Learning, PC – Autism-Related Parent Concerns. The Any 1, Any 2, or All 3 definitions includes infants whose scores met behavior marker definitions on at least one, at least two, or all three of the measures.
